# Integrated *in silico* identification of cholinesterase inhibitors from *Nyctanthes arbor-tristis*

**DOI:** 10.1371/journal.pone.0328457

**Published:** 2025-07-23

**Authors:** Qazi Mohammad Sajid Jamal, Ali H. Alharbi, Varish Ahmad, Khurshid Ahmad

**Affiliations:** 1 Department of Health Informatics, College of Applied Medical Sciences, Qassim University, Buraydah, Saudi Arabia; 2 King Salman Center for Disability Research, Riyadh, Saudi Arabia; 3 Health Information Technology Department, The Applied College, King Abdulaziz University, Jeddah, Saudi Arabia; Guru Nanak College, INDIA

## Abstract

Alzheimer’s disease (AD) remains a major neurodegenerative disorder, characterized by cognitive decline alongside functional impairments that affect daily living. Natural therapeutic alternatives have spurred the search for novel inhibitors targeting acetylcholinesterase (AChE) and butyrylcholinesterase (BChE), two well-established targets in AD therapy. This study utilizes an *in silico* methodology to investigate new cholinesterase inhibitors sourced from *Nyctanthes arbor-tristis,* a plant known for its abundant bioactive phytochemicals. Through virtual screening, molecular dynamics simulations (MDS), free energy landscape analysis, and ADMET predictions, eight compounds have been identified as potential inhibitors of AChE and/or BChE. These compounds include Apigenin, Arborside-B, Arbortristoside-D, Arbortristoside-E, Nicotiflorin, Arborside-A, Beta-amyrin, and Nyctanthic acid. These compounds exhibited robust binding affinities compared to the control, advantageous ADMET profiles, and sustained stability throughout 200 ns of MDS and energy assessments, highlighting their potential as viable drug candidates for further exploration. Further experimental studies are required to validate their therapeutic efficacies, thereby confirming their potential as neurological therapeutics for the treatment of AD and related disabilities.

## Introduction

Neurological disorders, such as Alzheimer’s disease (AD), epilepsy, seizures, brain aneurysms, meningitis, memory loss, muscular dystrophy, multiple sclerosis, Parkinson’s disease, migraines, strokes, encephalitis, and chronic headaches, affect millions of individuals. AD presents a considerable challenge owing to its progressive neurodegenerative characteristics and restricted treatment alternatives [1]. The key therapeutic strategy for managing AD involves the inhibition of acetylcholinesterase (AChE) and butyrylcholinesterase (BChE), enzymes responsible for the hydrolysis of acetylcholine, leading to improved cholinergic transmission [2,3]. Inhibiting these enzymes enhances cholinergic transmission by augmenting the availability of acetylcholine (Ach) in the synaptic cleft, thereby improving cognitive function, particularly in learning and memory [4]. AChE inhibitors, including rivastigmine, donepezil, and galantamine, are frequently prescribed for the management of mild to moderate AD. AChE primarily governs acetylcholine degradation in the central nervous system, whereas BChE gains significance in the advanced stages of AD, rendering dual inhibition a potentially effective therapeutic strategy [5,6].

AChE inhibitors (AChEIs) mitigate synaptic damage in AD by inhibiting the degradation of ACh, thereby enhancing cholinergic transmission in the brain [7,8]. AChE may facilitate the aggregation of amyloid-beta (Aβ), resulting in the production of toxic oligomers. AChE inhibitors serve a dual purpose in AD management by maintaining cholinergic function and potentially mitigating amyloid pathology. Moreover, the cholinergic system regulates neuroinflammation, a crucial element in cerebral impairment during severe illness, highlighting its extensive function in neuroprotection [9,10].

Neurotherapeutics of natural origin have been recovered from many medicinal plants and reported as a potential source of AChE inhibitors due to their safe bioactivity and structural diversity [11]. Among these *Nyctanthes arbor-tristis (N. arbor-tristis)* (night-flowering jasmine), a medicinal and ornamental plant traditionally used in Ayurvedic medicine. This plant, native to South Asia, has been widely consumed for its therapeutic properties, including antimicrobial, anti-inflammatory, antimalarial, and antioxidant activities, due to its reservoir of many bioactive compounds, such as glycosides, flavonoids, phenolic acids, and alkaloids, which exhibit multifaceted therapeutic effects [12–14]. *N. arbor-tristis* extracts have been shown in preclinical studies to modify neuroinflammatory pathways and decrease amyloid-beta toxicity, which are essential features of AD pathology [15,16]. Moreover, the bioactive molecules of this plant have been explored for their antioxidant properties that can regulate oxidative stress, a key driver of brain cell inflammation and neuronal damage in AD [17]. Researchers have tested flavonoids in the plant, such as phenolic acids and quercetin, for a variety of therapeutic uses. Therefore, in search of neurotherapeutics, this research aimed to screen the most interactive bioactive (AchE inhibitors) chemicals of this plant with the choline receptors, which can protect the breakdown of acetylcholine and ameliorate cognitive impairments associated with neurodegenerative diseases [18,19]. Exploring *N. arbor-tristis* as a source of natural AChE inhibitors provides a promising avenue for developing safer and more effective treatments for neurological disorders.

Prior computational investigations have effectively employed molecular docking and MD simulations to discern prospective natural inhibitors of AChE and BChE [20–22]. Nevertheless, the distinctive phytoconstituents of *N. arbor-tristis* remain predominantly unidentified in this context. This study uses molecular docking, MD simulations, and FEL analysis to investigate the conformational stability and binding dynamics of key bioactive compounds from *N. arbor-tristis*. This study assesses the phytoconstituents of *N. arbor-tristis* as potential dual inhibitors of AChE and BChE, utilizing donepezil as a benchmark. Donepezil, an AChE inhibitor with moderate BChE activity, is a pertinent benchmark for evaluating the selected natural compounds’ binding affinity and inhibitory efficacy [23].

## Methods

### Receptor and natural compounds preparation

Protein Data Bank (PDB) [24] was used to retrieve the crystal structure of human AChE (PDB ID: 7E3H) [25] and BChE (PDB ID: 7AIY) [26]. The heteroatoms, water molecules, and co-crystallized ligands were removed. Donepezil was selected as the reference compound because the selected AChE structure is co-crystallized with donepezil, allowing precise identification of binding interactions and providing a validated framework for molecular docking and virtual screening analyses. A total of 27 potential compounds from *N. arbor-tristis* has been identified in our previous works and other literature [14,27]. The PubChem database [28] was utilized to obtain the chemical structures of the selected bioactive compounds from *N. arbor-tristis* in SDF format, which were subsequently converted to 3D format using the PyRx tool [29] for docking purposes, employing the MMFF94 force field for energy minimization.

### Virtual screening

Molecular docking-based virtual screening was performed on 27 phytocompounds from *N. arbor-tristis* including control drug Donepezil against the active sites of AChE and BChE using AutoDock Vina [30] integrated into the PyRx tool. The co-crystallized ligand-binding site of AChE and BChE was considered a receptor-ligand interaction site. A grid box size of 60 × 60 × 60 Å³ was used to encompass the binding pocket. The grid was centered at coordinates (−43.369, 37.729, −30.314) for AChE and (−20.100, −14.859, 41.447) for BChE. Protein structures were prepared by removing water molecules and heteroatoms, and ligands were energy-minimized using Open Babel in PyRx. Post-docking visualization and interaction analysis, including hydrogen bonding, hydrophobic contacts, and π–π stacking, were performed using Discovery Studio Visualizer.

### Physicochemical and ADMET estimation

The ADMET attributes of the selected potential candidates were anticipated using the ADMET-AI web tool (https://admet.ai.greenstonebio.com). This tool employs machine learning models developed from comprehensive datasets to produce swift and dependable predictions of pharmacokinetic and toxicity parameters. Furthermore, it offers summary plots for an integrated assessment and radial plots to concurrently visualize multiple ADMET properties, thereby enabling a thorough evaluation of drug-likeness and safety profiles [31]. The platform generates ADMET radar plots, allowing visual interpretation of multiple properties such as absorption, distribution, metabolism, excretion, and toxicity, in a single integrated view.

### Molecular dynamics simulation (MDS)

The docked complexes underwent a 200 ns MDS utilizing GROMACS 2022. The CHARMM27 force field was applied for the protein, while ligand topology parameters were generated via SwissParam [32] to ensure compatibility. TIP3P water molecules were used to solve a cubic simulation box containing the complex and a 10 Å buffer. The system was neutralized with counterions (Na ⁺ /Cl⁻), and 0.15 M NaCl was added to simulate physiological conditions. The steepest descent algorithm was used to minimize energy (50,000 steps or until the force converged to less than 10.0 kJ/mol/nm). Equilibration was performed in two stages: NVT equilibration (100 ps, 300 K) utilizing the V-rescale thermostat with positional restraints on the receptor and solvent, followed by NPT equilibration (100 ps, 1 bar) employing the Parrinello-Rahman barostat to stabilize density.

The 200 ns production run was conducted with a 2-fs time step using periodic boundary conditions (PBC). The LINCS algorithm constrained bond lengths, while Particle Mesh Ewald (PME) was utilized for long-range electrostatics with a 10 Å cutoff for short-range interactions. Post-simulation analysis utilized GROMACS tools, encompassing RMSD (gmx rms), RMSF (gmx rmsf), Radius of Gyration (Rg, gmx gyrate), and Hydrogen Bond Analysis (gmx hbond). Results were illustrated utilizing Xmgrace for two-dimensional plots [33].

### MMPBSA calculation

Binding free energy (ΔGbind) was calculated using the MM-PBSA method via the gmx_MMPBSA tool [34] and MMPBSA.py script [35]. The components of ΔGbind included molecular mechanics energy (ΔEMM), solvation-free energy (ΔGsol), and entropic contribution (TΔS). This analysis provided thermodynamic insights into protein-ligand binding.

### Free energy landscape-based PCA analysis

Principal Component Analysis (PCA) was used to assess the conformational dynamics of protein-ligand complexes in MD simulations. Backbone Cartesian coordinates were extracted with GROMACS, and covariance matrices were computed to identify dominant motions represented by principal components (PC1 and PC2). The Free Energy Landscape (FEL) was created using PC1 and PC2 as reaction coordinates, with Gibbs free energy calculations identifying low-energy stable states. FEL plots were visualized with Python tools and showed stable regions in blue (low energy) and unstable regions in red (high energy), providing information about ligand stability and dynamic behavior.

## Results and discussion

Everyone, from individuals to their families and communities, is impacted by the adverse effects of AD on quality of life. Alzheimer’s patients may experience feelings of frustration, isolation, compounded by confusion, and exacerbated by the stigma often associated with cognitive disabilities. To manage these through natural phytochemicals, the 28 selected phytocompounds from *N. arbor-tristis* were subjected to molecular docking-based virtual screening against the active sites of AChE and BChE. The docked complexes were evaluated based on binding energy and interaction profiles, including hydrogen bonding and hydrophobic contacts. The top five compounds for each target, exhibiting the most favorable binding affinities, were selected for in-depth analysis, including ADMET prediction, MD simulations, and energy minimization, to assess their pharmacokinetic properties, stability, and binding dynamics.

Here, a detailed analysis of the top five compounds for AChE and BChE is presented, including their binding interactions, pharmacokinetic properties (ADMET), MD simulations, and energy minimization. This comprehensive evaluation provides insights into their stability, binding efficiency, and potential as dual cholinesterase inhibitors.

### Molecular docking analysis

**Fig 1:**
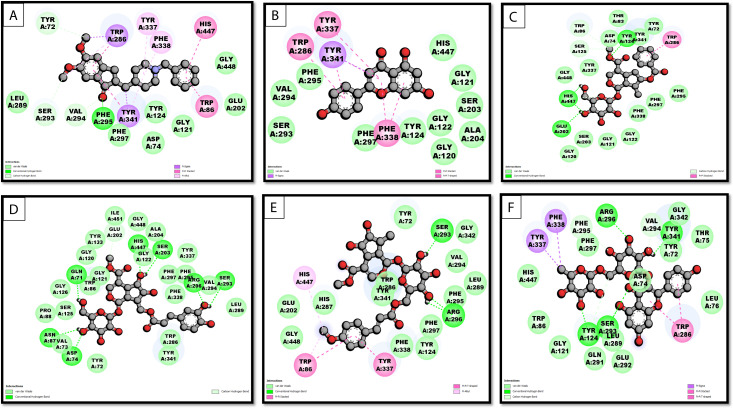
2D representation of AChE interaction with A) Donepezil (control); B) Apigenin; C) Arborside-B; D) Arbortristoside; E) Arbortristoside-E; F) Nicotiflorin.

**Fig 2:**
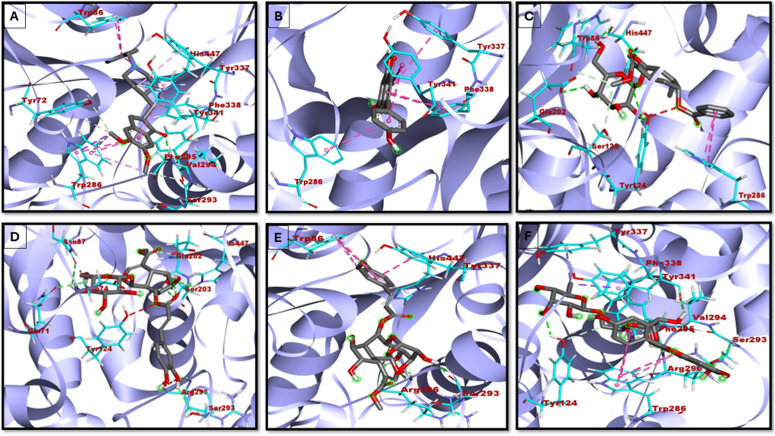
3D representation of AChE interaction with A) Donepezil (control); B) Apigenin; C) Arborside-B; D) Arbortristoside; E) Arbortristoside-E; F) Nicotiflorin.

The molecular interaction analysis of the top 5 compounds revealed that all the ligands showed significant binding energies with the AChE receptor, comparable to and close to the control drug, donepezil (−12.2 kcal/mol) was observed to interact with multiple hydrogen bonds with key amino acid residues like PHE295, VAL294, and TYR72. Additional interactions, including π-Sigma (TRP286, TYR341) and π-π stacking (HIS447, TRP86), reinforce its strong binding at the active site. The interactions align with key AChE residues, particularly the catalytic triad (HIS447, SER203) and peripheral anionic site Among the selected chemicals, Apigenin (−10.0 kcal/mol), Arborside-B (−9.9 kcal/mol), Arbortristoside-E (−10.0 kcal/mol), Arbortristoside-D (−9.8),and Nicotiflorin (−10.1 kcal/mol) exhibited promising interactions (Figs 1 and 2).

Figs 1 and 2 present 2D and 3D interaction analyses of the top 5 phytocompounds with AChE, compared to donepezil, the reference inhibitor. The docking results highlight key hydrogen bonding, hydrophobic interactions, and π-π stacking that contribute to ligand stability within the AChE active site. Overall, the 3D representations (Fig 2) confirm the stability of ligand binding observed in 2D interaction maps (Fig 1). The detailed results of interactions, including binding energy, hydrogen bonds, hydrogen bond lengths, van der Waals interactions, and other key interactions, are summarized in Table 1.

**Table 1:** AutoDock tool output data during the interaction of selected compounds and ACHE.

S.NO	Compound name	Binding energy (Kcal/mol)	H-bonds	H-bond length (Å)
1.	Donepezil (control)	−12.2	A:PHE295:HN - A:E20601:O24	1.7972
			A:VAL294:HA - A:E20601:O24	3.03571
			A:E20601:C26 - A:SER293:O	3.59102
			A:E20601:C28 - A:TYR72:OH	3.54587
2.	Apigenin	−10.0	NA	NA
3.	Arborside-B	−9.9	A:TYR124:HH -:UNK1:O13	2.68942
			A:HIS447:HE2 -:UNK1:O24	2.63996
			:UNK1:H57 - A:HIS447:O	2.29838
			:UNK1:H58 - A:GLU202:OE1	2.39545
			A:HIS447:HD2 -:UNK1:O21	1.82692
			A:HIS447:HD2 -:UNK1:O24	3.0709
			:UNK1:C14 - A:TRP86:O	3.66171
			:UNK1:C14 - A:SER125:OG	3.68176
4.	Arbortristoside-D	−9.8	A:ASP74:HN -:UNK1:O39	2.07253
			A:ARG296:HN -:UNK1:O27	1.91413
			A:HIS447:HE2 -:UNK1:O29	2.61473
			:UNK1:H70 - A:GLN71:OE1	2.82837
			:UNK1:H71 - A:ASN87:OD1	2.67555
			:UNK1:H60 - A:SER293:OG	2.79009
			:UNK1:H62 - A:SER203:OG	2.78321
			:UNK1:C1 - A:GLU202:OE1	2.49379
5.	Arbortristoside-E	−10.0	A:ARG296:HN -:UNK1:O34	2.166
			:UNK1:H71 - A:SER293:O	1.86012
			:UNK1:H69 - A:ARG296:O	2.22894
6.	Nicotiflorin	−10.1	A:TYR124:HH -:UNK1:O41	2.32
			A:SER293:HN -:UNK1:O28	2.84081
			A:SER293:HG -:UNK1:O27	2.91732
			A:ARG296:HN -:UNK1:O38	2.52697
			:UNK1:H67 - A:TYR341:O	2.00319
			A:VAL294:HA -:UNK1:O37	2.31041

**Fig 3:**
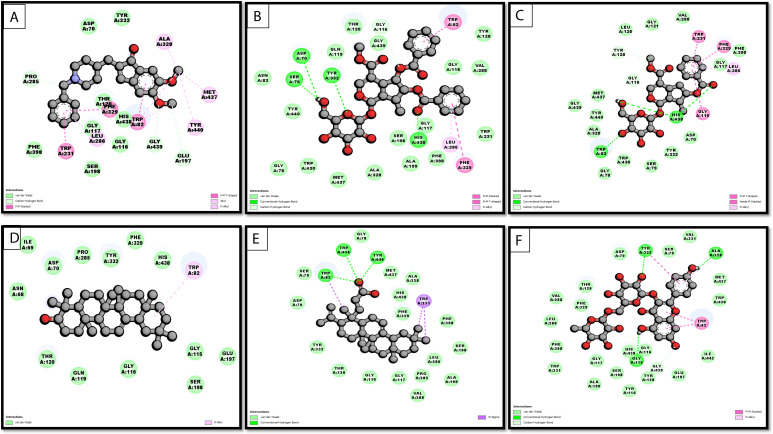
2D representation of BChE with A-Donepezil; B-Arborside-A; C-Aroborside-B; D- beta amyrin; E-Nyctanthic acid; F- Nicotiflorin.

**Fig 4:**
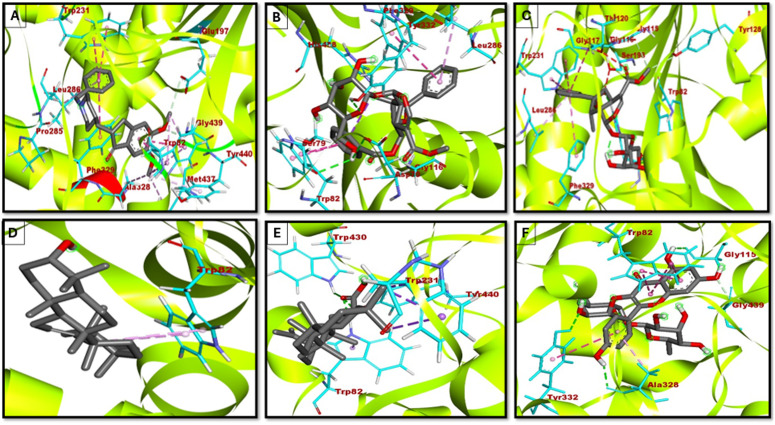
3D representation of BChE with A-Donepezil; B-Arborside-A; C-Aroborside-B; D- beta amyrin; E-Nyctanthic acid; F- Nicotiflorin.

Similarly, the docking analysis of the top five phytocompounds against BChE (Figs 3 and 4, Table 2) revealed strong binding affinities, with binding energies ranging from −9.8 to −11.0 kcal/mol, suggesting stable protein-ligand complex formation.

**Table 2:** Docking details for each compound with BChE, including compound name, binding energy, hydrogen bonds, and hydrogen bond lengths.

	Compound name	Binding energy (kcal/mol)	H-bonds	H-bond length (Å)
1.	Donepezil (control)	−9.8	A:GLY439:HA2 -A:E20601:O27	2.73269
			A:E20601:C17 - A:PRO285:O	3.31605
			A:E20601:C28 - A:GLU197:OE1	3.54874
2.	Arborside-A	−10.3	A:TYR332:HH -:UNK1:O35	2.37217
			A:HIS438:HE2 -:UNK1:O9	1.79906
			:UNK1:H72 - A:ASP70:OD1	2.8352
			:UNK1:H72 - A:SER79:O	2.78722
			A:GLY116:HA1 -:UNK1:O16	2.45639
3.	Arborside-B	−10.7	A:TRP82:HE1 -:UNK1:O24	2.30602
			A:HIS438:HE2 -:UNK1:O29	2.16106
			:UNK1:H57 - A:HIS438:O	2.43014
			:UNK1:C14 - A:GLY115:O	3.54479
			:UNK1:C14 - A:TYR128:OH	3.67164
4.	Beta-amyrin	−10.7	NA	NA
5.	Nyctanthic_acid	−11.0	A:TRP82:HE1 -:UNK1:O14	2.47765
			A:TRP430:HE1 -:UNK1:O14	2.94217
			A:TYR440:HH -:UNK1:O14	2.52418
6.	Nicotiflorin	−10.8	A:TYR332:HH -:UNK1:O37	2.63646
			:UNK1:H66 - A:ALA328:O	2.26199
			:UNK1:H60 - A:GLY115:O	2.37994
			A:GLY439:HA1 -:UNK1:O29	2.62847

Control molecule (−9.8 kcal/mol) demonstrated strong hydrogen bonding (GLY439, GLU197, PRO285), π-π stacking (TRP82, PHE329, and TRP231), and hydrophobic interactions with TYR440, MET437, and ALA328, indicating its stability in the active site (Figs 3A and 4A). Arborside-A shows binding energy −10.3 kcal/mol and formed hydrogen bonds (TYR332, HIS438, ASP70, SER79, and GLY116) (Figs 3B and 4B). Arborside-B (−10.7 kcal/mol) and Beta-amyrin (−10.7 kcal/mol) showed strong binding, with Arborside-B forming hydrogen bonds (TRP82, HIS438, GLY115) and Beta-amyrin engaging in extensive hydrophobic interactions (Figs 3C, 3D and 4C, 4D). Among the analysed compounds, Nyctanthic acid (−11.0 kcal/mol) showed the highest binding affinity, forming multiple hydrogen bonds (TRP82, TYR440, TRP430), π-stacking (TRP231), and hydrophobic contacts with ASP70, TYR332, THR120, and PHE329 (Figs 3E and 4E). Nicotiflorin (−10.8 kcal/mol) showed comparable stability, forming hydrogen bonds (TYR332, ALA328, GLY439), π-π stacking (TRP82, TYR332), and hydrophobic interactions with ALA328, PHE329, and MET437, indicating favorable binding (Figs 3F and 4F). The Nicotiflorin and Arborside-B have been identified as dual inhibitors against AChE and BChE.

### MD simulation

**Fig 5:**
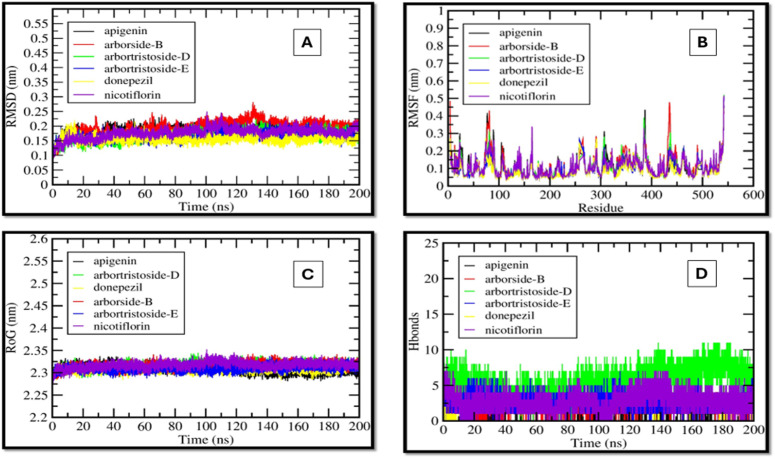
MD simulation analysis of AChE complexes with Donepezil and selected compounds over 200 ns. (A) RMSD, (B) RMSF, (C) Rg, and (D) H-Bonds.

Further, the MD simulation data of the AChE complexes with the selected five hits and the control drug were analyzed based on Root Mean Square Deviation (RMSD), Root Mean Square Fluctuation (RMSF), Radius of Gyration (Rg), and hydrogen bond profiles. The RMSD analysis (Fig 5A) elucidates the stability of protein-ligand complexes throughout the 200 ns simulation. Control compound exhibited a low RMSD (0.15–0.2 nm), signifying considerable stability. Nicotiflorin and Apigenin demonstrated comparable RMSD values (0.2 nm), indicating stable binding. Arbortristoside-E and Arborside-B exhibited moderate stability with RMSD values of approximately 0.25–0.3 nm, whereas Arbortristoside-D demonstrated the highest RMSD of around 0.3–0.35 nm, signifying increased conformational flexibility. Compounds with elevated docking scores (e.g., Nicotiflorin, Apigenin) demonstrated reduced RMSD, thereby substantiating their robust and stable interactions with AChE.

The RMSF values provide residue-level flexibility across the simulation, as shown in Fig 5B. Higher fluctuations were observed around loop regions and non-active site residues. The amino acid residues within the binding pocket, including TYR124, TRP286, HIS447, and TYR341, displayed minimal fluctuations for all compounds, suggesting stable ligand-protein interactions. Arbortristoside-D showed slightly higher fluctuations, which aligns with its higher RMSD and indicates reduced binding stability. The compounds forming stronger hydrogen bonds (e.g., Nicotiflorin, Apigenin) showed lower flexibility in binding site residues, reinforcing their stable docking poses.

The Rg values reflect the compactness of the protein during the simulation, which is shown in Fig 5C. All complexes analyzed maintained a stable Rg (2.3–2.35 nm), which signified that the protein structure remained compact throughout the simulation. Donepezil showed the lowest Rg fluctuations, while Arbortristoside-D exhibited slightly higher variations, suggesting some destabilization of the protein structure. The stability of Rg for Nicotiflorin and Apigenin supports their strong binding interactions observed in docking.

Hydrogen bonds (H-bonds) are critical for ligand binding stability, as shown in Fig 5D. Donepezil formed the highest number of consistent H-bonds (15–20), ensuring strong binding throughout the simulation. Similarly, Nicotiflorin and Apigenin maintained 10–15 H-bonds, indicating stable binding. Arborside-B and Arbortristoside-E: formed 8–12 H-bonds, showing moderate stability. Arbortristoside-D: exhibited the lowest H-bond count (5–8), correlating with its higher RMSD and reduced stability. Compounds with higher binding energies and extensive docking interactions (e.g., Nicotiflorin, Apigenin) formed more H-bonds during MD simulations (Fig 5D).

Many phytochemicals from the alkaloids, flavonoids, terpenoids, and phenolic compounds have been reported to exhibit AChE inhibitory activity [11]. The MD results align well with the docking data, reinforcing the potential of *N. arbor-tristis* compounds as AChE inhibitors: Nicotiflorin and Apigenin consistently exhibited strong binding stability, supported by low RMSD, minimal RMSF, stable Rg, and higher H-bond counts. Their docking profiles showed extensive Pi-Pi stacking and hydrophobic interactions, which MD validated. Arborside-B and Arbortristoside-E showed moderate binding stability, consistent with their slightly lower docking scores. Arbortristoside-D exhibited the least stability, correlating with its higher RMSD and fewer H-bonds in MD. The findings confirm the promising inhibitory potential of Nicotiflorin and Apigenin, with performance comparable to donepezil.

**Fig 6:**
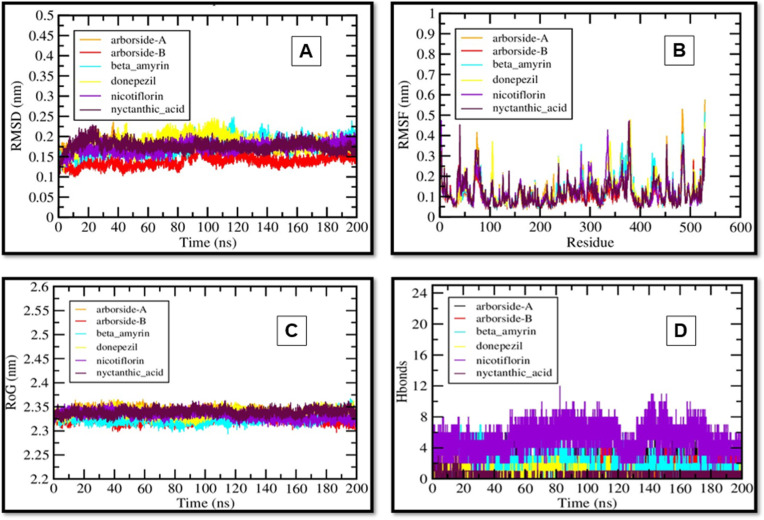
MD simulation analysis of BChE complexes with Donepezil and selected compounds over 200 ns. (A) RMSD, (B) RMSF, (C) Rg, and (D) H-Bonds.

Further, the MD simulation of BChE complexes was also assessed. The RMSD analysis shows that all protein-ligand complexes have stabilized over time, confirming the convergence of MD simulations (Fig 6A). Donepezil (yellow) and Nicotiflorin (purple) had the lowest RMSD values (0.1–0.2 nm), indicating high structural stability. Arboroside-A and Arboroside-B had slightly higher RMSD (0.2–0.25 nm), indicating moderate flexibility, while Nyctanthic acid showed initial fluctuations before stabilization. As expected, RMSF analysis showed minimal fluctuations (<0.4 nm) in most residues, with higher fluctuations in terminal and loop regions. Nicotiflorin and Nyctanthic acid had consistently low RMSF values, indicating minimal dynamic perturbations and strong protein-ligand interactions (Fig 6B).

Rg values (2.3–2.5 nm) were stable, indicating compact protein-ligand complexes. Donepezil and Nicotiflorin had the lowest Rg values (2.3–2.35 nm), indicating well-folded and stable complexes. Arboroside-B and Nyctanthic acid had slightly higher values (Fig 6C). Hydrogen bond analysis revealed that all compounds had stable interactions (8–12 H-bonds), contributing to complex stability. Nicotiflorin and Nyctanthic acid consistently form moderate hydrogen bonds (4–12), indicating a strong binding affinity. Arborosides A and B formed fewer hydrogen bonds, indicating weaker interactions (Fig 6D).

### PCA analysis

**Fig 7:**
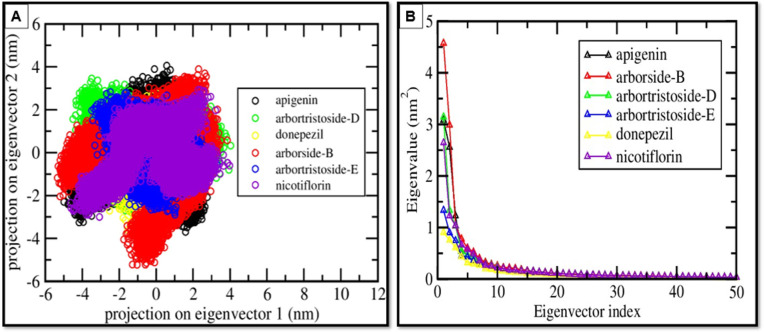
PCA of protein dynamics. (A) Projection of motion on the first two principal components for the top 5 compounds and control bound to AChE compared to the control. (B) Eigenvalue distribution plot showing the contribution of each eigenvector to the total motion.

The 2D PCA projection of AChE complexes emphasizes the conformational space explored by protein-ligand complexes. Nicotiflorin (purple) and Donepezil (yellow) exhibit compact clusters, signifying stable dynamics with restricted conformational variability. Arbortristoside-B (red), Arbortristoside-D (green), Arbortristoside-E (blue), and Apigenin (black) exhibit more dispersed distributions, signifying enhanced flexibility and transient binding. The eigenvalue analysis reveals a swift decrease following the initial eigenvectors, indicating that the primary principal components capture the bulk of the conformational variance. Nicotiflorin and donepezil exhibit lower eigenvalues, signifying reduced large-scale motion and enhanced stability. Arbortristoside-D and analogous compounds display higher eigenvalues, suggesting increased flexibility that may affect alternative protein conformations (Fig 7).

**Fig 8:**
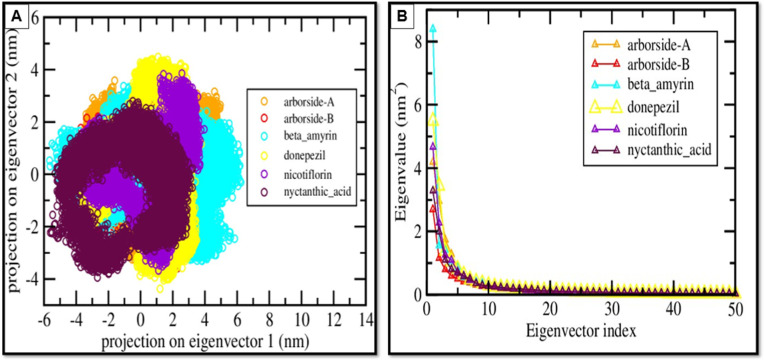
PCA of protein dynamics. **(A)** Projection of motion on the first two principal components for the top 5 compounds and control bound to BChE compared to the control. **(B)** Eigenvalue distribution plot showing the contribution of each eigenvector to the total motion.

Similarly, the 2D PCA projections of BChE complexes (Fig 8A) indicate that Donepezil (yellow) and Nicotiflorin (purple) form compact clusters, which suggests limited conformational variability and high stability. In contrast, Arborside-A, Arborside-B, Beta-amyrin, and Nyctanthic Acid exhibited broader distributions, suggesting increased conformational flexibility. The eigenvalue analysis (Fig 8B) demonstrates a significant decrease in eigenvalues following the initial eigenvectors, implying that most conformational variance is encapsulated in the first principal components. Nicotiflorin and donepezil demonstrate lower eigenvalues, which implies diminished large-scale motion and increased stability. In contrast, Arborside-A, Arborside-B, and Beta-amyrin show higher eigenvalues, reflecting enhanced flexibility.

### MMPBSA analysis

Furthermore, MMPBSA calculations of AChE complexes show that Arbortristoside-D (−41.89 kcal/mol) has the highest binding affinity, outperforming donepezil (−21.66 kcal/mol), indicating more stable interactions with AChE. Arborside-B (−23.48 kcal/mol), Arbortristoside-E (−24.53 kcal/mol), and Nicotiflorin (−23.04 kcal/mol) all exhibit stronger binding than donepezil, indicating their potential as inhibitors. Apigenin (−12.67 kcal/mol) has the weakest binding, making it a less desirable candidate. Donepezil (−266.13 kcal/mol) has the most potent gas-phase interactions, followed by Arbortristoside-D (−98.34 kcal/mol) and Arbortristoside-E (−86.91 kcal/mol). Apigenin has the weakest ΔG_GAS (−71.09 kcal/mol), indicating its low affinity. Donepezil (244.47 kcal/mol) has the highest solvation penalty (ΔG_SOLV), resulting in a lower net binding affinity. In contrast, Arbortristoside-D (56.45 kcal/mol) and Arbortristoside-E (62.53 kcal/mol) have a better balance, which improves their stability. Low SEM values (e.g., 1.16 for Arbortristoside-D, 1.10 for Nicotiflorin) demonstrate statistical reliability and consistent measurements. Arborside-B (SEM = 2.40) exhibits slightly more variability. Overall, Arbortristoside-D emerges as the most promising candidate. Arborside-B, Arbortristoside-E, and Nicotiflorin also show potential for further optimization (Table 3).

**Table 3:** Representing the data of Poisson Boltzmann-based free binding energy calculation of the Ligand-AChE receptor energy components.

Compounds	ΔGGAS	ΔGSOLV	ΔGTOTAL	SD	SEM
Donepezil (Control)	−266.13	244.47	−21.66	1.49	1.49
apigenin	−71.09	58.42	−12.67	0.75	1.54
Arborside-B	−74.08	50.59	−23.48	0.81	2.40
arbortristoside-D	−98.34	56.45	−41.89	0.91	1.16
Arbortristoside-E	−86.91	62.53	−24.53	2.45	1.60
nicotiflorin	−81.59	58.55	−23.04	2.02	1.10

SD – Sample standard deviation, SEM – Sample standard error of the mean.

Further, the MMPBSA binding energy calculations of BChE complexes (Table 4) showed that Nicotiflorin (−33.46 kcal/mol) had the highest binding affinity, significantly outperforming donepezil (−22.44 kcal/mol). Nyctanthic Acid (−31.44 kcal/mol) exhibited superior binding, while Arborside-A (−25.78 kcal/mol) and Arborside-B (−24.69 kcal/mol) demonstrated slightly stronger binding than donepezil. Beta-amyrin (−21.50 kcal/mol) had the weakest binding, indicating a limited potential as a BChE inhibitor.

**Table 4:** Representing the data of Poisson Boltzmann-based free binding energy calculation of the Ligand-BChE receptor energy components.

Compounds	ΔGGAS	ΔGSOLV	ΔGTOTAL	SD	SEM
Donepezil (Control)	−143.30	120.86	−22.44	1.84	1.50
Arborside-A	−82.32	56.54	−25.78	0.98	2.24
Arborside-B	−78.88	54.19	−24.69	0.33	1.28
Beta-amyrin	−61.93	40.43	−21.50	1.28	1.46
Nyctanthic acid	−51.97	20.53	−31.44	1.19	0.82
nicotiflorin	−129.81	96.34	−33.46	3.35	1.02

(SD – Sample standard deviation, SEM – Sample standard error of the mean).

Donepezil had the strongest gas-phase interactions (ΔGGAS) (−143.30 kcal/mol), followed by Nicotiflorin (−129.81 kcal/mol), indicating significant van der Waals and electrostatic contributions. Nyctanthic Acid had the weakest gas-phase interaction (−51.97 kcal/mol). However, its low solvation penalty (ΔGSOLV = 20.53 kcal/mol) increased its overall binding energy. Donepezil had the highest solvation penalty (120.86 kcal/mol), lowering its net binding affinity, whereas Nicotiflorin (−96.34 kcal/mol) and Nyctanthic Acid (−20.53 kcal/mol) retained strong binding stability due to lower solvation costs.

### FEL analysis

**Fig 9:**
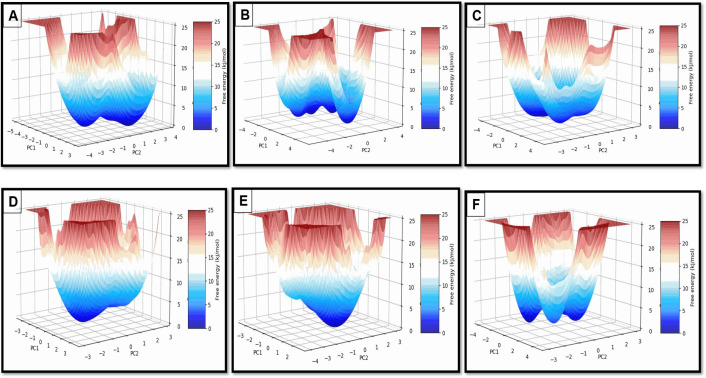
3D FEL plots of top selected AChE complex with compounds. A) apigenin; B) Arborside-B; C) arbortristoside-D; D) Arbortristoside-E; E) donepezil; F) nicotiflorin.

The FEL plots of AChE complexes (Fig 9, Panels A–F) depict the conformational dynamics and stability of AChE complexes with the chosen compounds. The FEL, derived from PCA, condenses conformational space into two principal components (PC1 and PC2), with free energy values represented on the z-axis denoting favored conformational states and thermodynamic stability.

Apigenin and donepezil demonstrate singular deep energy wells, signifying highly stable conformations with negligible fluctuations, thereby reinforcing their robust binding interactions. Arbortristoside-E forms a stable complex with a somewhat shallower potential well, indicating moderate stability. Arborside-B and Nicotiflorin exhibit extensive, multi-basin energy wells, indicating enhanced conformational flexibility, presumably attributable to weaker or adaptable interactions. Arbortristoside-D exhibits two separate energy minima, indicating dynamic transitions between stable conformational states that may influence binding kinetics. In conclusion, donepezil demonstrates excellent stability, consistent with its potent AChE inhibitor function. Apigenin, Arbortristoside-E, and Nicotiflorin exhibit moderate stability, whereas Arborside-B and Arbortristoside-D display enhanced flexibility, potentially affecting their inhibitory efficacy. These insights are crucial for rational drug design, emphasizing candidates with stable binding dynamics and distinctive conformational behaviors (Fig 9).

**Fig 10:**
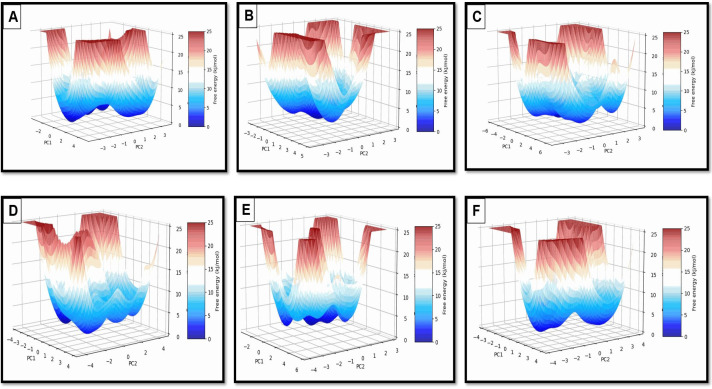
3D FEL plots of top selected BChE complex with compounds. **A)** arborside-A; **B)** Arborside-B; **C)** beta amyrin; **D)** Donepezil; **E)** nyctanthic acid; **F)** nicotiflorin.

The 3D FEL plots in Fig 10 reveal information about the thermodynamic stability of BChE-ligand complexes. Donepezil has a deep, narrow energy well, indicating high stability and low conformational flexibility, consistent with its potent inhibitor role. Beta-amyrin has two deep energy wells, indicating strong binding and limited flexibility. Arborside-B and Nyctanthic Acid, on the other hand, have broader, shallower energy wells, indicating greater flexibility but less stability. Nicotiflorin and Arborside-A have dual energy minima, indicating conformational adaptability, which may increase inhibitory efficacy.

### ADMET evaluation

**Fig 11:**
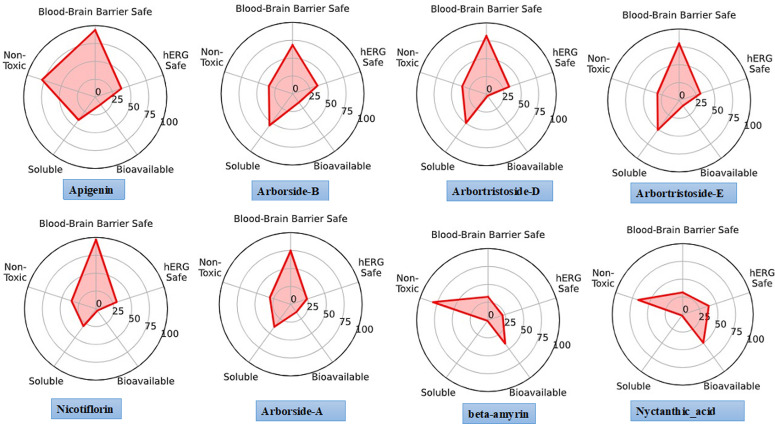
Radar plots of ADMET properties for selected AChE and BChE inhibitors using the ADMET-AI web tool.

An extensive interaction study was conducted on eight distinct compounds, derived from 10 top-ranked candidates (five for AChE and five for BChE, with two common), and subsequently assessed for their ADMET properties to evaluate drug-likeness and potential for drug discovery. The ADMET radar plots (Fig 11) show key pharmacokinetic and toxicity parameters, confirming that all compounds are BBB-permeable and, thus, potential CNS-active drugs for AD treatment.

Apigenin, Nicotiflorin, and Nyctanthic Acid are among the selected compounds with high bioavailability and solubility, making them more drug-like and suitable for oral administration. The majority of compounds are nontoxic and hERG-safe, which reduces the risk of cardiotoxicity. However, beta-amyrin and some Arborside derivatives have a lower solubility, which may affect absorption and bioavailability. Nicotiflorin, Apigenin, and Nyctanthic Acid are the most promising candidates, with excellent pharmacokinetic properties, high binding affinities, and stability. Their favorable ADMET profiles indicate their potential for further experimental validation and optimization as dual AChE and BChE inhibitors.

## Conclusion

This study demonstrates that compounds derived from *N. arbor-tristis* can function as dual inhibitors of AChE and BChE, offering a safer and more sustainable alternative for managing AD and cognitive decline by exhibiting robust binding and favorable drug-like properties. Apigenin, Beta-amyrin, and Nicotiflorin emerged as the most promising candidates, exhibiting high binding affinities, advantageous ADMET properties, and central nervous system permeability. The FEL analysis demonstrated distinct conformational flexibility, especially in Beta-amyrin and Nicotiflorin, suggesting their potential for targeting multiple therapeutic applications. This study computationally validates the neurological therapeutic relevance of these compounds in comparison to donepezil. At the same time, identifying understudied phytochemicals, such as Arborside-A and Nyctanthic Acid, expands the scope of neurodegenerative research. This research establishes a foundation for integrating natural product chemistry into contemporary drug discovery, facilitating economical, multi-target therapies for AD, and managing cognitive decline. Nevertheless, the absence of experimental validation is one of the study’s limitations. Although the *in silico* results are encouraging, additional *in vitro* and/or *in vivo* research is required to ascertain the identified compounds’ safety profiles and cholinesterase inhibitory activity. Biochemical experiments and biological analysis will be the primary focus of future research to verify these computer predictions and advance the identified candidates toward therapeutic applicability.

## Supporting information

S1 Data Data-set. (ZIP)
